# Effectiveness of Different Virtual Reality Technologies for Social and Communication Skills in Children With Autism Spectrum Disorder: Systematic Review and Network Meta-Analysis of Current Evidence and Future Directions

**DOI:** 10.2196/82814

**Published:** 2026-04-30

**Authors:** Lin Wang, Guangjun Xu, Dan Li, Xiuyan Gao, Jin Zhao, Yajun He, Shaolong Liu, Hong Guo, Xiumei Bu

**Affiliations:** 1 Liaoning University of Traditional Chinese Medicine, No. 79, Chongshan Road, Huanggu District, Shenyang, Liaoning Province, China, 86 13940036489, 86 024-31207206; 2 Liaoyang Vocational College of Technology, Liaoyang, Liaoning Province, China; 3 Health Service Center of Liaoning Province, Shenyang, Liaoning Province, China; 4 Liaoning Nursing Association, Shenyang, Liaoning Province, China; 5 Shenyang Medical College, Shenyang, Liaoning Province, China

**Keywords:** autism spectrum disorder, virtual reality, social communication skills, network meta-analysis, children

## Abstract

**Background:**

Virtual reality (VR) technology offers a new approach for the intervention of social communication skills in children with autism spectrum disorder (ASD), but the comparative effects of different forms of VR technology remain unclear.

**Objective:**

This study aims to conduct a systematic review and network meta-analysis (NMA) based on existing randomized controlled trials (RCTs) to initially explore and compare the effects of different VR technologies on improving the social and communication skills of children with ASD.

**Methods:**

We systematically searched relevant RCTs in both Chinese and English databases from January 1990 to February 2025. The quality of the literature was evaluated using the revised Cochrane risk of bias assessment tool (RoB-2), and an NMA was conducted under the frequentist framework using STATA 18.0 software. The quality of evidence was assessed using the Confidence in Network Meta-Analysis framework.

**Result:**

A total of 11 RCTs (718 children) were included, evaluating 8 VR technologies. The evidence network was extremely sparse, with most interventions connected by single studies. Pairwise meta-analysis revealed overwhelming heterogeneity (*I*²=91.9%, *P*<.001), indicating profound clinical and methodological diversity. Due to this heterogeneity and the sparse network, the NMA model failed to produce stable or clinically interpretable effect estimates. Formal assessment using the Confidence in Network Meta-Analysis framework rated the confidence in all comparisons as very low.

**Conclusions:**

The existing evidence is insufficient to support any comparative efficacy conclusions or rankings among VR technologies for ASD social skills. The key finding is the demonstration that current evidence is too heterogeneous and immature for valid quantitative synthesis. Future research must prioritize methodological standardization before head-to-head trials can be meaningfully conducted.

## Introduction

Autism spectrum disorder (ASD), commonly referred to as autism, is a neurodevelopmental disorder that originates in early childhood. Its primary characteristics include impairments in social interaction and communication, repetitive patterns of behavior, and restricted interests or activities [1]. The global prevalence of autism is on the rise [2]. According to the 2023 report from the US Centers for Disease Control and Prevention, by 2020, the prevalence of ASD among 8-year-old children was approximately 1 in 36 (4% for boys and 1% for girls), which is higher than the estimation of the Autism and Developmental Disabilities Monitoring network from 2000 to 2018 [3]. A multistage convenience cluster sampling study in China indicates that the estimated prevalence of ASD among children aged 6 to 12 years in China is 0.70%, equivalent to approximately 700,000 children [4]. One of the core symptoms of ASD is social impairment, which manifests as a lack of early social interest and motivation compared to peers, ultimately affecting their ability to engage socially [5]. Even when they exhibit social interest, they often lack the necessary social skills for appropriate interaction with others [6]. This leads to difficulties in effectively communicating and interacting with others, hindering their ability to maintain normal social relationships, which further impacts their language skills and mental health [7]. Therefore, it is essential to identify effective and sustainable measures to enhance social and communication skills among individuals with ASD.

Although current medical research has made certain progress, the exact cause of the disease has not been fully clarified. Existing studies suggest that the disorder may be caused by the interaction of multiple factors, such as genetic susceptibility [8], environmental exposure [9], and abnormal changes in the neurodevelopmental process. Due to the complexity of its pathogenesis, there is currently no specific treatment strategy targeting the cause [10]. Currently, clinical practice primarily employs comprehensive treatment, encompassing drug therapy, behavioral modification, educational training, and physical therapy [11,12]. However, traditional treatments still have some limitations. For example, behavioral training relies heavily on the therapeutic room environment and lacks ecological validity in real-world social scenarios, making it difficult to transfer skills [13]. Second-generation antipsychotic drugs (such as risperidone) can alleviate aggressive behavior but cannot improve core symptoms and carry the risk of metabolic syndrome [14]. In addition, autism is characterized by a high disability rate and currently lacks an effective cure. Its management primarily involves long-term, intensive professional rehabilitation interventions aimed at enhancing the overall abilities of children with autism. Nevertheless, these interventions impose significant economic burdens, demand substantial time commitments from caregivers, and exert considerable psychological pressure [15]. Moreover, they present formidable challenges for the allocation of social public resources, the establishment of professional service systems, and ensuring their long-term sustainability.

Since the 1990s, numerous empirical studies have systematically explored the feasibility and effectiveness of utilizing virtual reality (VR) for training and intervention in individuals with ASD [16]. VR technology is capable of integrating the real and virtual worlds, replicating diverse scenarios via algorithms, generating immersive experiences, and enabling human-computer interaction through controllers, thereby embodying the characteristics of immersion, interactivity, and imagination [17]. Over the past 2 decades, VR has been extensively applied in medicine and has garnered increasing attention in clinical cognitive rehabilitation [18]. Relevant research indicates that VR not only enhances the life skills of individuals with ASD [19], but also improves their cognitive abilities [20], emotional regulation and recognition skills [21], as well as social and communication competencies [22]. Studies demonstrate that VR technology exhibits unique advantages in addressing core symptoms in children with ASD through mechanisms, such as neuroplastic remodeling, behavioral reinforcement learning, and multimodal compensation [23].

Current VR intervention studies encompass desktop-based, augmented reality, immersive, and hybrid technologies [24], with intervention content spanning areas, such as social communication and emotional cognition [25]. However, the evidence base is characterized by profound heterogeneity in intervention protocols, outcome measurement instruments, and participant characteristics. Moreover, head-to-head comparisons between different VR modalities are virtually absent, and the existing studies primarily compare each active intervention against heterogeneous control conditions. This fragmented evidence landscape renders conventional pairwise meta-analysis insufficient for comparative efficacy questions, but it also raises fundamental concerns about whether the more complex network meta-analysis (NMA) can be validly applied.

While NMA offers a theoretical framework to integrate direct and indirect evidence and derive comparative effect estimates even when head-to-head trials are lacking, its validity critically depends on the assumptions of transitivity and consistency. Given the anticipated clinical and methodological diversity among studies in this nascent field, these assumptions are likely to be violated.

Therefore, we aim to (1) systematically map the existing randomized controlled trial (RCT) evidence on VR interventions for social and communication skills in children with ASD; (2) formally assess whether the current evidence base satisfies the assumptions required for a valid NMA; (3) assess if these assumptions are seriously violated, to conduct a detailed methodological “autopsy” to characterize the sources and magnitude of heterogeneity, network sparsity, and evidence gaps; and (4) derive concrete, prioritized recommendations for future research that address the identified methodological barriers. By reframing the analysis from hypothesis verification to hypothesis generation and from comparative efficacy assessment to evidence readiness assessment, this study aims to provide a rigorous foundation for the design of future comparative effectiveness trials and for the eventual translation of VR technologies into clinical practice.

## Methods

This systematic review was conducted in accordance with the PRISMA (Preferred Reporting Items for Systematic Reviews and Meta-Analyses) and the PRISMA extension of the NMA guidelines [26], the details of which can be found in Checklist 1. This study is registered in the PROSPERO (International Prospective Register of Systematic Reviews) international systematic evaluation platform (CRD420250654696).

### Search Strategy and Inclusion and Exclusion Criteria

The selection and search strategies for eligible studies were constructed based on the PICOS (population/patient, intervention, comparator, outcome, and study design) framework. We systematically retrieved data from 8 electronic databases (PubMed, Embase, Cochrane Library, Web of Science, EBSCOhost, CNKI, VIP, and Wanfang) from 1990 to February 26, 2025. To ensure no eligible literature was overlooked, we also examined the reference lists of earlier systematic reviews [23,27-30] and the included studies as supplementary sources. Due to the limitations of obtaining professional resources, only literature published in both Chinese and English was included in this search. The detailed search strategy is introduced in Multimedia Appendix 1. Following a thorough search of numerous databases, duplicate publications were discarded. Titles and abstracts were then screened, and full texts were assessed according to the inclusion and exclusion criteria. The screening and selection processes were independently conducted by 2 evaluators (L.W and XG). Any differences are determined through consultation by a third evaluator (XB).

Table 1 displays the specific selection criteria. Overall, if a study meets the following conditions, it is considered to be eligible: (1) the trial design is an RCT aiming to evaluate the effectiveness of any VR intervention on children with autism; (2) recruitment of children diagnosed with ASD is based on clinical assessments or the criteria from the *DSM-5* (*Diagnostic and Statistical Manual of Mental Disorders, Fifth Edition*) or other recognized diagnostic standards (such as the *Autism Diagnostic Observation Schedule, 2nd Edition* or the *International Classification of Diseases, 10th Edition*); (3) participants in the control group underwent non-VR interventions, nondrug treatments, or routine nursing care, whereas those in the experimental group were exposed to VR interventions; and (4) at least 1 outcome related to social or communication function is reported in the outcome indicators. Studies will be excluded if they (1) are republished articles; (2) cannot provide the full text or have a high risk of bias (such as an unrigorous trial design, lack of participant data, etc); or (3) are reviews, observational studies, case reports, letters to the editor, or conference abstracts.

**Table 1.** PICOS (population/patient, intervention, comparator, outcome, and study design) criteria for inclusion of studies.

Parameter	Criteria
Population	Children and adolescents under 18 years of age and were diagnosed with ASD^a^
Intervention	Research involving any type of virtual reality intervention
Comparator	No limitations on the control group except virtual reality interventions, such as no-treatment, waiting-list control, traditional care, or cannot be included in other treatment nodes
Outcomes	Any outcomes regarding social and communication skills that can be measured
Study design	Randomized controlled trials

a ASD: autism spectrum disorder.

### Outcome

The primary outcome was social and communication skills. The efficacy was expressed as the change in the overall social and communication symptom assessment score after the VR intervention (data collected before and after the intervention).

### Data Extraction

Two independent reviewers (XG and SL) extracted relevant information in a standard manner, including bibliographic data (author, publication year, and country/region), participant characteristics (age, gender, and sample size), intervention components (category, frequency, and duration), and immediate postintervention primary outcome measures. In cases where studies used 2 or more measurements for the same outcome indicator, the task most commonly utilized was included. If a single task had multiple raw scores, higher-quality results were preferred. The formula from the Cochrane Handbook was used to calculate the changes in mean and SD relative to the baseline when they were not fully reported [31]. We reached out to the corresponding author via email to gather more information if any data were missing. The Cochrane risk of bias tool for randomized trials [32] was used to assess the methodological quality of the included RCTs. The evidence quality of social and communication abilities was evaluated within the framework of CINeMA (Confidence in Network Meta-Analysis) [33].

For the purpose of this NMA, interventions were grouped based on their primary technological interface as reported in the original studies. Given the significant variation in specific hardware, software, and intervention protocols even within the same broad category and the diversity of control conditions, we explicitly acknowledge that these operational groups may encompass substantial clinical and methodological heterogeneity. This heterogeneity is a critical consideration when interpreting the transitivity assumption of the NMA and the pooled results, as discussed in the *Limitations* section.

### Data Analysis

The data analysis was conducted jointly by 2 researchers (LW and DL). Given the significant clinical and methodological differences among the included studies in terms of population characteristics, intervention protocols, outcome measures, and the sparse preliminary evidence network, this study adopts a hierarchical analysis strategy. First, we will conduct a detailed descriptive synthesis, systematically presenting and comparing the key features and main findings of each study. Subsequently, we will perform an exploratory NMA to visualize the evidence structure and generate preliminary, hypothetical comparative results. It must be emphasized that due to the aforementioned limitations, the point estimates and ranking results of the NMA have high uncertainty and should be regarded as a hint for future research directions rather than definitive efficacy conclusions or clinical recommendation bases.

All data analyses were performed using STATA 18.0 (StataCorp LLC) software, following the protocol outlined below.

All outcomes were continuous variables. To mitigate baseline discrepancies, effect sizes were pooled using changes in mean values and SDs before and after the intervention. Given the variability in assessment tools and units across studies, standardized mean differences (SMDs) were adopted as the effect metric. First, traditional pairwise meta-analyses were conducted using the “metan” command to compute pooled SMDs and their 95% CIs for all comparisons between VR interventions and usual care, with forest plots generated for visualization. A random-effects model was employed to account for between-study heterogeneity, which was quantified using the *I*² statistic: *I*²≤50% indicated low heterogeneity, whereas *I*²>50% denoted high heterogeneity. Transitivity was evaluated by comparing the distribution of study characteristics across intervention comparison pairs—specifically, examining whether characteristics were balanced across all intervention pairs connected via a common comparator. Systematic differences in characteristic distributions would suggest the potential violation of the transitivity assumption. We compared the distribution of key covariates that might affect the treatment effect between the groups in direct comparison and found no obvious systematic imbalance. This provides a preliminary basis for transitivity in the network analysis. However, due to the limited number of studies, the assessment of this assumption still needs to be cautious. In fact, given the observed clinical diversity in participants, interventions, and outcomes across studies, we anticipated potential violations of these assumptions. Therefore, all NMA results are presented as exploratory estimates, and the ranking of interventions is interpreted with caution, emphasizing the generation of hypotheses for future research rather than definitive clinical conclusions.

Building on descriptive analyses, exploratory NMA was conducted. Evidence networks were visualized using the “network” command to illustrate direct and indirect comparisons among distinct VR technologies. For closed loops within the network, node-splitting was performed to test for consistency; in cases of inconsistency, an inconsistency model was applied [34]. The analysis model was fitted using the “mvmeta” command under a frequentist framework, which allows sharing of a common heterogeneity parameter across comparisons. The surface under the cumulative ranking curve was calculated using the “sucra” command to generate preliminary rankings of interventions [35]. League tables summarizing SMDs and 95% CIs for all pairwise intervention comparisons were produced using the “netleague” command. Funnel plots were generated via the “metafunnel” command, and the Egger test (implemented via the “metabias” command) was used to quantitatively assess small-study effects [36]. Leave-one-out sensitivity analysis was conducted using the “metaninf” command to evaluate the stability of pooled effect sizes by sequentially excluding each study. To explore potential sources of heterogeneity, subgroup analyses were performed based on predefined factors (eg, intervention modality, geographic region, and intervention duration). Additionally, meta-regression was conducted using the “metareg” command to examine the association between continuous variables (eg, sample size and total intervention length) and effect sizes. All analyses were conducted using a 2-tailed test, with the significance level *α* set at 0.05.

## Results

### Summary of Results

**Figure 1.**
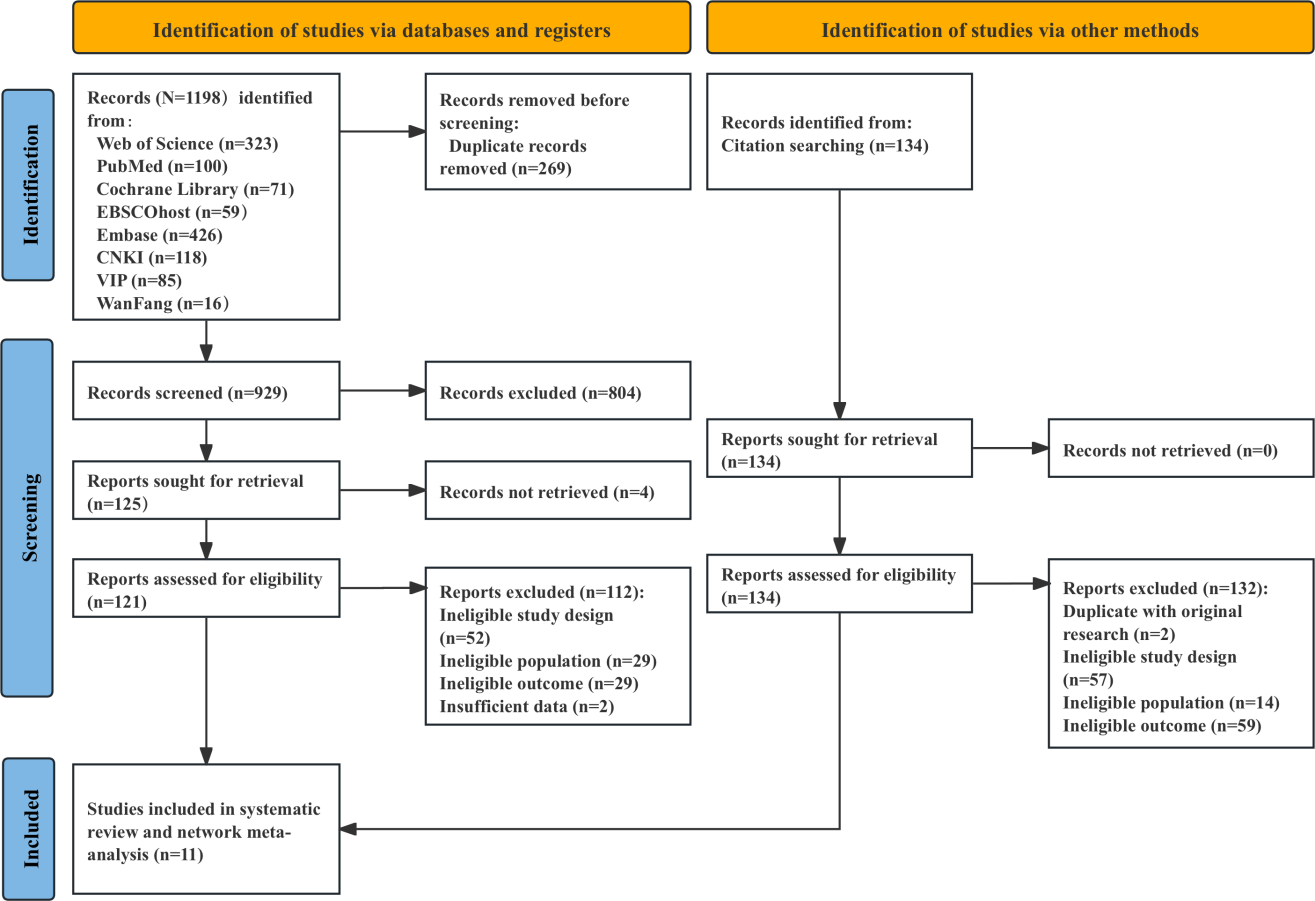
PRISMA (Preferred Reporting Items for Systematic Reviews and Meta-Analyses) flowchart of study selection.

The retrieval of the system identified 1198 records from the electronic database. Once duplicates were eliminated, the titles and summaries of the bar records were reviewed, and 125 full-text articles were obtained to assess their eligibility. Another 134 records determined from the reference list of the relevant systematic review were also screened as qualified. The method used for literature screening is presented in Figure 1. Finally, a total of 11 studies [37-47] were included in this review, involving 718 children with ASD.

However, the preliminary synthesis of direct comparison evidence revealed extremely high heterogeneity among the studies (*I*²=91.9%, *P*<.001). This result suggests that there are fundamental differences in intervention protocols, participant characteristics, and outcome measurement tools among the various studies, which make the traditional combined effect size insufficiently robust and clinically meaningful when interpreting the overall efficacy. Therefore, the following analysis will focus on describing the current state of evidence and exploratory findings.

### Research Characteristics

Table 2 summarizes the details of each included study. Among the 11 studies, all employed recognized diagnostic criteria for participant identification. The age of participants ranged from preschoolers to adolescents. The experimental interventions comprised 8 distinct forms of virtual technology, classified as digital platform, head-mounted display (HMD), VR glasses, mixed reality, CAVE (cave automatic virtual environment), Half-CAVE, desktop VR, and computer-based magic skill training. HMD was the most frequently evaluated technology (n=3), followed by digital platforms. Control conditions varied and included conventional rehabilitation care, wait-list controls, or other active non-VR therapies.

**Table 2.** Characteristics of included studies.

Study (year)	Country	Diagnostic criteria	Sample size		Sex		Age (y)		Treatment	Protocol details		Length	Duration per session	Frequency	Main outcome index
			E^a^	C^b^	E	C	E	C		E	C				
Wang [37] (2024)	China	DSM-5^c^	30	30	—^d^	—	3-5	3-5	Digital platform^e^	Immersive virtual reality (VR) (HLKF-DT-01 platform): nine modules including (1) attention (piano keys, basketball), (2) language shadowing (progressive sentence repetition), (3) spatial orientation (virtual classroom/street navigation), (4) daily-living rehearsal	Conventional rehabilitation—group OT^f^, ADL^g^ training, language therapy via Orff music, family-guided outdoor play, and social-story practice	4 weeks	20 min	5 days/week	ABC^h^
Zhao et al [38] (2021)	China	DSM-5	57	57	—	—	3-5	3-5	HMD^i^	Home-based VR (HMD and smartphone app): identical 9-module curriculum with added (1) affect-expression tasks (avatar facial mimicry), (2) fine/gross-motor tracking games (gesture-based)	Home rehabilitation care—daily scenario education, balanced diet/exercise plans, parent-mediated play, token-economy reward system	6 months	20 min	2 sessions/week	ABC
Voss et al [39] (2019)	America	DSM-5	40	31	37M^j^/3F^k^	16M/15F	Mean 8.63 (SD 2.52)	Mean 8.74 (SD 1.79)	Superpower Glass^l^	Wearable artificial intelligence (AI) system (Superpower Glass): Google Glass and emotion-recognition Convolutional neural network; provides (1) peripheral green box for face detection, (2) emoji and audio cue for 8 emotions	Home rehabilitation care—applied behavior analysis (ABA); therapist-delivered ABA at home; discrete trial training, naturalistic teaching	6 weeks	20 min	4 sessions/week	VABS-II^m^
Sayis et al [40] (2022)	Spain	ADOS^n^ module 3	36	36	30M/6F	30M/6F	8-12	8-12	MR^o^	Mixed reality floor projection (6-m diameter): cooperative firefly-catching game triggering virtual characters; light emitting diode net tracking and multicamera motion capture	Conventional rehabilitation—LEGO cooperative play; therapist-guided dyadic construction of pirate ship; hexagonal table setup; verbal prompting for social initiation	Once	15 min	—	ASS^p^
Yuan and Ip [41] (2018)	Hong Kong, China	DSM-5	36	36	31M/5F	33M/3F	Mean 8.97 (SD 1.10)	Mean 8.73 (SD 1.15)	CAVE^q^	CAVE projection system: six authentic scenarios—(1) morning routine, (2) bus ride, (3) library rules, (4) tuck-shop conflicts, (5) playground consolidation	Wait-list control—no VR or structured social skills intervention during the study period	6 weeks	60 min	1 session/week	PEP-3^r^
Zhao et al [42] (2022)	China	DSM-5	22	22	19M/3F	16M/6F	3-4	3-4	HMD	Unity3D VR scenes: 6 modules—object search, color sorting, animal interaction; AI scaffolding: target-highlight	Conventional rehabilitation—group oral instruction, sensory-integration equipment (balance boards and tactile balls)	12 weeks	15 min	3 sessions/week	PEP-3
Jiang et al [43] (2023)	China	DSM-5	31	31	20M/11F	19M/12F	Mean 13.47 (SD 1.23)	Mean 13.87 (SD 1.08)	HMD	VR eye-tracking (J2-R2-1020): gaze-contingent dialogue initiation; saccade-triggered virtual character interaction (120 Hz sampling, <0.5° calibration)	Conventional rehabilitation—oral vitamin D₃, sand-play therapy twice weekly, no digital component	6 months	30 min	3 sessions/week	ATEC^s^
Ip et al [44] (2018)	Hong Kong, China	DSM-5	36	36	31M/5F	33M/3F	Mean 8.97 (SD 1.11)	Mean 8.74 (SD 1.15)	H-CAVE^t^	4-side CAVE projection: six social-emotion scenarios with (1) relaxation environment and (2) school rule practice	Wait-list control—standard school curriculum plus usual outpatient therapy; no VR exposure	14 weeks	—	2 sessions/week	PEP-3
Ye et al [45] (2020)	China	DSM-5	32	32	19M/13F	20M/12F	Mean 3.51 (SD 1.03)	Mean 3.54 (1.05)	Computer^u^	VR-SST^v^ platform: avatar-mediated role-play (greeting, sharing); AI immediate feedback	Conventional rehabilitation—traditional SST; therapist-led role-play, feedback, homework; 30-min sessions covering peer interaction, emotion expression	3 months	30 min	3 sessions/week	ABC
Wang et al [46] (2016)	China	DSM-4	35	35	30M/5F	29M/6F	Mean 4.23 (SD 1.63)	Mean 3.91 (SD 1.44)	Digital platform	Dolphin House AV system: 2–8 kHz bionic dolphin sounds and 3D ocean VR and rhythmic lighting (0.5–4 Hz); tactile plush dolphin vibration. Acoustic intensity: 60–75 dB; illuminance 200–400 lux	Conventional rehabilitation—table-top social stories, token reinforcement, therapist-guided play; no digital component	6 months	45 min	15 days/phase	ABC
Yuen et al [47] (2023)	America	DSM-5	9	8	7M/2F	7M/1F	Mean 12.3 (SD 2.3)	Mean 10.5 (SD 1.2)	Computer	Virtual magic training via Zoom: OT-student coaches teach 2–3 tricks/session (cards, rubber bands, and ropes); Hocus Focus Evaluation Scale; mailed prop kit	Wait-list control—1-month delay before identical virtual MTTP^z^; no active intervention during control phase	3 weeks	45 min	3 sessions/week	SSIS^x^

a E: experimental group.

b C: control group.

c DSM-5: Diagnostic and Statistical Manual of Mental Disorders, Fifth Edition.

d Not available.

e Digital platform: Digital evaluation interactive training platform.

f OT: occupational therapy.

g ADL: activities of daily living.

h ABC: Autism Behavior Checklist.

i HMD: head-mounted display.

j M: male.

k F: female.

l Superpower Glass: Google Glass works with smartphones.

m VABS-II: Vineland Adaptive Behavior Scales Second Edition.

n ADOS module 3: Autism Diagnostic Observation Schedule, Module 3.

o MR: mixed reality.

p ASS: Self-made questionnaire: Affective Slider scales.

q CAVE: cave automatic virtual environment.

r PEP-3: Psychological Educational Profile - Third Edition.

s ATEC: Autism Treatment Evaluation Checklist.

t H-CAVE: Half Cave Automatic Virtual Environment.

u Computer: Desktop VR common equipment.

v VR-SST: virtual reality-based social skills training.

w MTTP: magic trick training program.

x SSIS: Social Skills Improvement System.

Notably, there was substantial diversity in the intervention protocols. Even within the same technology category, the specific content of virtual scenarios, interaction modalities, session duration, intervention frequency, and total intervention period differed markedly across studies. This indicates that each study investigated a unique “intervention package” rather than a standardized application of a given technology.

### Evidence Network and Assessment of Heterogeneity

**Figure 2.**
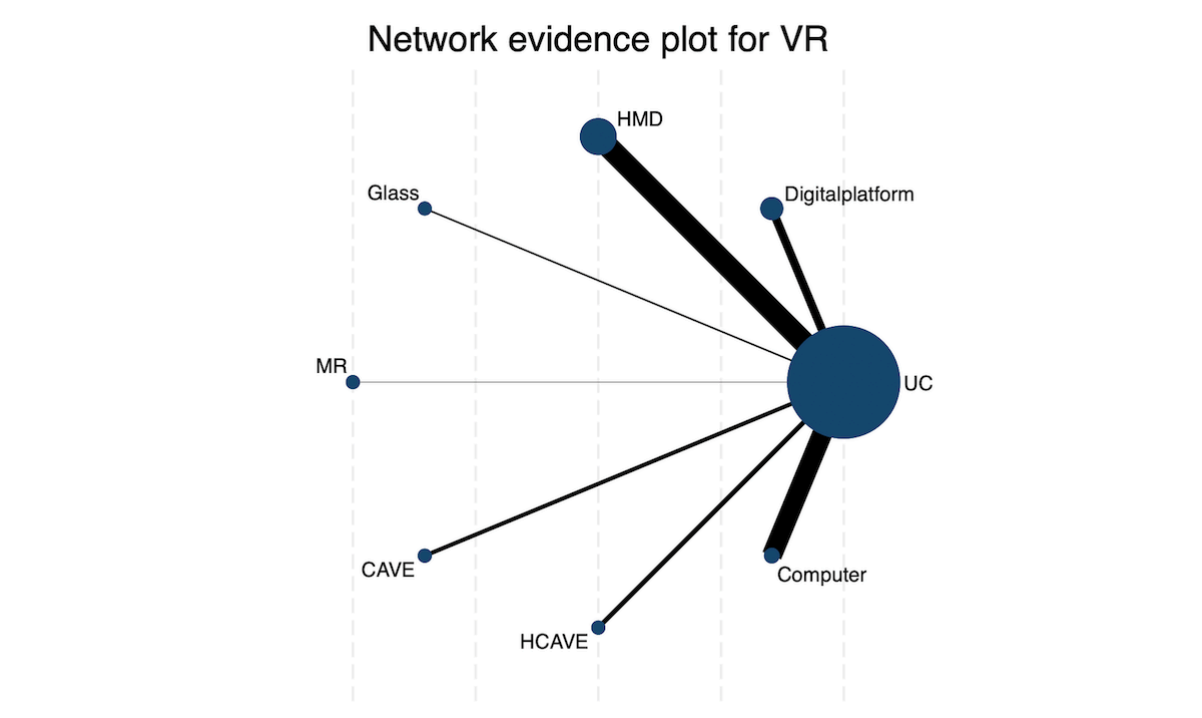
Network of eligible comparisons for the primary outcome (social and communication skills). CAVE: cave automatic virtual environment; H-CAVE: half cave automatic virtual environment; HMD: head-mounted display; MR: mixed reality; VR: virtual reality.

Figure 2 presents the geometry of the treatment network for the primary outcome. The network is sparse and unbalanced. While several direct comparisons exist between HMD and conventional care (forming the backbone of the network), many other intervention nodes are connected by only a single study. A large number of potential comparisons between different active VR technologies lack direct evidence and must rely entirely on indirect estimations. This sparsity fundamentally limits the stability and reliability of any quantitative comparative estimates derived from the network.

The preliminary pairwise meta-analysis of all VR interventions versus control groups revealed an exceptionally high degree of statistical heterogeneity (*I*²=91.9%, *P*<.001). This overwhelming heterogeneity is not merely statistical but reflects profound clinical and methodological diversity across studies in terms of participant profiles, the nature and intensity of VR interventions, and—most critically—the tools used to measure social and communication skills. These tools assess different constructs and dimensions of social functioning with varying sensitivity. Consequently, the traditional pooled effect size, while indicative of a general positive direction, is too heterogeneous to be meaningfully interpreted as a single, precise estimate of efficacy.

### Exploratory Quantitative Synthesis Findings

Given the severe and anticipated violations of the NMA assumptions—demonstrated by the extreme statistical heterogeneity (*I*²=91.9%, *P*<.001) and the sparse, disconnected evidence network—any quantitative synthesis must be interpreted with the highest degree of caution.

Under a frequentist random-effects framework, the model produced comparative effect estimates for all contrasts; however, the 95% CIs were implausibly wide, and the model failed to achieve stable convergence. The leave-one-out sensitivity analysis confirmed that no single study was responsible for these extreme outputs; rather, the instability is an intrinsic mathematical consequence of synthesizing highly heterogeneous studies within an inadequately connected network. The formal assessment of the confidence in the evidence using the CINeMA framework rated all comparisons as “very low,” driven by serious concerns regarding within-study bias, intransitivity, imprecision, and network sparsity (see Multimedia Appendix 1).

Consequently, we do not report any specific SMDs, CIs, or surface under the cumulative ranking curve values in this section. These unstable numerical outputs are provided in Multimedia Appendix 1 for transparency and reproducibility purposes only. They must not be cited, interpreted, or used to infer the comparative efficacy or rank order of the included VR technologies. The sole robust quantitative finding from this analysis is the *I*² statistic of 91.9%, which unequivocally demonstrates that the included studies are too clinically and methodologically diverse to be meaningfully combined for the purpose of comparative effect estimation.

### Risk of Bias and Quality of Evidence

We used the revised Cochrane risk of bias tool (RoB-2) to assess the included studies, and the results are detailed in Multimedia Appendix 1. The overall risk of bias was judged as moderate. The analysis revealed a distinct pattern: the most prominent sources of bias pertained to the randomization process and deviations from intended interventions. In these domains, a notable proportion of studies were rated as “high risk,” accompanied by a substantial number with “some concerns,” marking them as the core contributors to the overall risk of bias. The selection of the reported result emerged as a prevalent area of potential bias; while fewer studies were “high risk” in this domain, the majority raised “some concerns,” indicating a widespread methodological limitation. In contrast, risks in the domains of measurement of the outcome and missing outcome data were relatively lower, with assessments predominantly being “low risk” and only sporadically “some concerns,” suggesting these aspects were better controlled.

Furthermore, we used the CINeMA framework to rate the confidence in the NMA evidence of social and communication skills, which was very low in all comparisons. The results are detailed in Multimedia Appendix 1. This rating was driven by serious concerns about within-study bias (moderate risk of bias in included trials), nontransitivity (clinical and methodological heterogeneity), imprecision (very wide CIs), and sparse data (a small number of studies forming the network). This very low confidence rating formally emphasizes the high uncertainty of the quantitative estimates and rankings.

### Other Exploratory Analyses

Meta-regression did not indicate the significant effects of region, intervention form, intervention duration, and intervention cycle on social and communication skills. Meanwhile, the subgroup analysis showed significant heterogeneity among different regions and different intervention form groups. The research results are presented in Multimedia Appendix 1. The source of this heterogeneity might be due to the insufficient sample size of the included original studies. Furthermore, in a single intervention, a treatment course of more than 40 minutes showed significant heterogeneity, which might be attributed to methodological differences, individual differences among participants, publication bias, among other factors. In Multimedia Appendix 1, the sensitivity analysis demonstrated that excluding studies with a high risk of bias generally yielded results consistent with the original findings. The funnel plot in Multimedia Appendix 1 shows that the scattered points are mainly located at the top of the funnel and demonstrated bilateral symmetry, indicating that the studies using the Social Functioning Assessment Scale as the outcome measurement have the least publication bias. However, 3 studies were located outside the funnel and were rather scattered, indicating that there might be a certain degree of publication bias, which could be due to the small sample size and low accuracy.

## Discussion

### Key Findings

This is the first study to apply the NMA to compare VR technologies for social skills in children with ASD. The most salient finding, however, is not a comparative effect estimate or ranking but rather a negative one: the existing evidence base is too heterogeneous, sparse, and methodologically inconsistent to support any valid quantitative synthesis. The critical findings are not comparative effect sizes or intervention rankings but rather (1) extreme and irreducible statistical heterogeneity (*I*²=91.9%, *P*<.001), reflecting profound clinical and methodological diversity; (2) a sparse and disconnected evidence network in which most intervention nodes are supported by single studies and the majority of pairwise comparisons lack any direct evidence; (3) clear violations of the transitivity and consistency assumptions required for a meaningful NMA; and (4) “very low” confidence in all comparative estimates as rated by the CINeMA framework. In essence, the attempt to perform an NMA “failed”—and this failure is itself the most robust and clinically informative result of this study. Therefore, this discussion will first critically examine these limitations and then, within the framework of the existing evidence, cautiously explore the potential value and challenges of different VR technologies and point out directions for future research.

### Why Did the NMA Fail? Limitations and Sources of Heterogeneity in the Existing Evidence

Although the NMA provides a theoretical framework for comparing multiple interventions, the reliability of its results depends on the internal consistency of the evidence base [48]. The extreme heterogeneity observed in this study (*I*²=91.9%) is not accidental but stems from fundamental, irreconcilable differences at multiple levels that directly violate the transitivity and consistency assumptions of NMA.

First, in terms of outcome measurement tools, various studies employed a range of tools, such as from the Autism Behavior Checklist and Psychoeducational Profile to the Vineland Adaptive Behavior Scales, each assessing social functions in different dimensions, with varying degrees of sensitivity and scoring methods. The direct combination of their data assumes conceptual equivalence that does not hold, and this alone renders any pooled effect size uninterpretable [49]. Second, in terms of intervention protocols, even within the category of “head-mounted displays,” there are significant differences in core elements, such as the specific content of virtual scenarios, interaction logic, duration of each session, total intervention period, and whether therapist guidance is included, making them essentially different “intervention packages.” Third, in terms of study subjects, key characteristics, such as the age range of children, severity of ASD, and cognitive function levels, vary. These clinical and methodological diversities make it difficult to directly compare the study results and are the main reasons for the wide range of effect sizes observed. These clinical and methodological diversities are not merely nuisances to be statistically adjusted; they represent fundamental violations of the assumption that studies are sufficiently similar to be synthesized for comparative inference. Consequently, the primary robust conclusion from this quantitative exercise is the profound inability of the existing data to support stable or credible comparative effect estimates using the NMA.

### The Potential Value and Implementation Considerations of Different VR Technologies

While acknowledging the aforementioned limitations, a descriptive synthesis of existing research can still offer valuable insights. Various forms of VR technologies, including HMDs, desktop VR, and augmented reality, have all reported positive improvements in social skills in their respective studies [50]. Among them, HMDs have shown outstanding potential in multiple studies due to their ability to provide highly immersive, controllable, and customizable virtual social environments [51]. This sense of immersion may help attract the attention of children with ASD and allow them to practice social interactions in a safe and highly repetitive environment, which aligns with the views presented in the systematic review by Bradley and Newbutt [52]. However, this does not imply that HMDs are superior to other modalities; they are simply the most intensively studied to date. For instance, desktop VR, with its lower sensory load and higher operational convenience, may be more suitable for some sensitive individuals or as an initial adaptation tool [53]. Meanwhile, CAVE systems, despite being limited to fixed locations, offer a unique shared space experience and are suitable for group training that requires close guidance from therapists [23].

At the same time, we must confront the practical challenges that VR intervention, especially immersive devices, faces in clinical translation. Although initial data suggest that children with ASD have a good acceptance of HMDs, the collection of safety data regarding sensory hypersensitivity, anxiety induction, or cybersickness is still neither systematic nor sufficient [54]. In addition, equipment costs, the need for professional technical support, and the integration of intervention programs with existing rehabilitation systems are all key obstacles to their wide promotion [55]. Future intervention frameworks should include a structured “transition from virtual to real” phase and actively explore the combination with mature paradigms, such as natural developmental behavior intervention [56].

### Implications for Future Research

Given the current weak and inconsistent evidence base, research in this field urgently needs to move from exploring feasibility to building high-level evidence. However, the path to high-level evidence does not begin with head-to-head trials; it begins with methodological standardization.

First and foremost, methodological standardization is an indispensable prerequisite. We strongly advocate that future research studies adopt a consensus-based core outcome set and report the specific parameters of the intervention protocol in detail to enhance comparability among studies. Without this foundational step, even large-scale comparative trials will remain nonsynthesizable and will not advance the field. Second, there is a need to design and implement head-to-head RCTs—but only after the above standardization has been achieved. Such trials should directly compare the efficacy of different VR technologies in the same population and with the same measurement tools, rather than only comparing them with passive control groups. However, until outcome measures and intervention descriptors are harmonized, the results of such trials will remain context-bound and difficult to replicate or generalize. Third, the research perspective needs to go beyond immediate effects and incorporate long-term follow-up evaluations to examine the retention and generalization of skills to real-world situations and systematically monitor and report adverse events. Finally, exploring personalized intervention matching based on individual characteristics will be an inevitable path to achieving precise rehabilitation.

In conclusion, VR offers a promising new toolkit for social skills intervention in autism. This review indicates that various forms of VR hold potential, but current research is still in its early stages, with limited and heterogeneous evidence quality. We cannot, and should not, claim any specific technology as the “best” choice based on the existing data. Future efforts should focus on strengthening the evidence base, improving technical solutions, and promoting the safe, effective, and equitable integration of VR into multimodal ASD intervention systems.

### Limitations

This study has several important limitations that must be fully taken into account when interpreting its results.

First, the sparsity of the evidence network is the fundamental factor that restricts the explanatory power of this NMA. Although 11 studies were included, there were as many as 8 intervention measures being compared, resulting in many comparison nodes being supported by only a single study. This “broad but shallow” evidence structure means that, for most comparisons between technologies, the effect estimates are highly dependent on indirect evidence, thereby increasing the instability and uncertainty of the results. The ranking probability results generated under this sparse network should be regarded as extremely preliminary exploratory hints rather than conclusive efficacy rankings.

Second, this study observed significant and unexplained heterogeneity. Although we attempted to explore the sources of heterogeneity through the subgroup analysis and meta-regression, the clinical and methodological diversity among studies in terms of population characteristics, intervention details, and core outcome measurement tools constituted irreducible systematic differences. Particularly, the standardization and combination of scale scores based on different theoretical constructs and measurement units, although a methodological convention, might have obscured the specific impact of the intervention on different dimensions of social function. Therefore, the large effect size intervals and fluctuations in the combined estimates reported mainly reflect this fundamental heterogeneity, suggesting that simple quantitative synthesis may not accurately describe the complex reality. In contrast, sensitivity analyses confirmed that the extreme effect estimates were an intrinsic product of the evidence structure and not the result of individual outliers. Thus, the core value of this study lies not in the numerical results it generates, but in the fact that it clearly reveals that current evidence is insufficient for reliable quantitative comparisons. Furthermore, the classification of interventions and comparators, while necessary for quantitative synthesis, itself introduces a source of heterogeneity. This clinical and methodological diversity directly challenges the similarity assumption required for a robust NMA and is a primary reason for the high statistical heterogeneity observed and the wide confidence intervals in our effect estimates.

Third, there may be language and search biases. To ensure the feasibility of the search, the language of the literature in this review was limited to Chinese and English, which might have resulted in missing relevant studies published in other languages, thereby affecting the comprehensiveness of the evidence base.

Fourth, the depth and breadth of outcome measures are insufficient. All included studies focused on the immediate postintervention effects and generally lacked medium- and long-term follow-up data, thus making it impossible to assess the sustainability of VR intervention effects and their generalization to real-life scenarios. Additionally, for the ASD population, which is sensitive to sensory stimuli, only a few studies systematically reported adverse reactions or reasons for dropout, leaving a gap in the comprehensive assessment of the safety profile of VR technology, especially immersive devices.

Finally, the risk of bias in the included studies should be treated with caution. Nearly half of the studies had “some concerns” or “high risk” in the randomization process or blinding implementation, which might have affected the internal validity of the effect estimates to some extent. Although the sensitivity analysis showed that the direction of the main conclusion remained unchanged after excluding the high-risk studies, this risk indicates that more methodologically rigorous studies are needed in the future to strengthen the evidence base.

### Conclusion

This is the first NMA to quantitatively compare diverse VR technologies for improving social and communication skills in children with ASD. The most salient finding is not about the superiority of any specific technology but rather about the current state of the evidence base: it is too limited, heterogeneous, and methodologically inconsistent to support reliable conclusions regarding comparative effectiveness.

The core value of this study lies in systematically reviewing the current evidence, highlighting the key gaps that need to be addressed and demonstrating that the evidence is not yet ready for comparative synthesis. Future research must make breakthroughs in the following areas: first, more high-quality, large-sample RCTs should be conducted, especially those directly comparing different VR technologies, to provide more reliable data on therapeutic efficacy. Second, efforts should be made to standardize outcome measurement tools and use consensus-based core sets of indicators to enhance comparability among studies and the accumulation of evidence. Third, long-term efficacy and safety must be emphasized, including the assessment of skill maintenance, generalization, and potential risks associated with technology use. Fourth, individualized intervention plans based on personal characteristics should be explored to determine which technologies are most suitable for different types of children with autism.

In conclusion, VR shows promising potential as an intervention tool, but its evidence base is still in its infancy. The current research findings should be regarded as generating hypotheses about which technologies merit further investigation, not as validating conclusions about their comparative effectiveness. We call on the academic community to work together to advance methodological harmonization as the essential foundation for all subsequent comparative research studies.

## Supplementary material

10.2196/82814 Multimedia Appendix 1 Search strategies, risk-of-bias assessment, exploratory quantitative synthesis, meta-regression, subgroup analysis, sensitivity analysis, funnel plot, and Confidence in Network Meta-Analysis assessment.

10.2196/82814 Checklist 1 PRISMA checklist.

## Abbreviations

ASD: autism spectrum disorder
CAVE: cave automatic virtual environment
CINeMA: Confidence in Network Meta-Analysis
DSM-5: Diagnostic and Statistical Manual of Mental Disorders, Fifth Edition
HMD: head-mounted display
NMA: network meta-analysis
PICOS: population/patient, intervention, comparator, outcome, and study design
PRISMA: Preferred Reporting Items Statement for Systematic Reviews and Meta-Analyses
PROSPERO: International Prospective Register of Systematic Reviews
RCT: randomized controlled trial
RoB-2: revised Cochrane risk of bias assessment tool
SMD: standardized mean difference
VR: virtual reality
