# Supplementary material for: Effectiveness of Different Virtual Reality Technologies for Social and Communication Skills in Children With Autism Spectrum Disorder: Systematic Review and Network Meta-Analysis of Current Evidence and Future Directions
Source: JMIR Pediatr Parent. 2026 Apr 30;9:e82814. doi: 10.2196/82814 (PMC13132534; doi:10.2196/82814)
Supplement: Multimedia Appendix 1 [file pediatrics-v9-e82814-s001.docx]

**Index of supplementary material**

Supplementary 1 Search strategy 2

Supplementary 2 Cochrane risk-of-bias assessment results 4

Supplementary 3 Exploratory Quantitative Synthesis Findings 5

Supplementary 4 Exploratory meta-regression and subgroup analysis 8

Supplementary 5 Exploratory sensitivity analysis 9

Supplementary 6 Exploratory funnel plot 10

Supplementary 7 Grading the evidence of the network meta-analysis using CINeMA 11

**Supplementary 1 Search strategy**

**Systematic Search Syntax for Electronic Databases (1990-February 2025)**

| **Pubmed** | |
| --- | --- |
| #1 | "Autism Spectrum Disorder"[MeSH] OR "autism" OR "ASD" OR "Autistic" OR "autistic disorder" OR "pervasive developmental disorder" OR "asperger syndrome" |
| #2 | "Virtual Reality"[MeSH] OR "VR" OR "virtual environment" OR "immersive technology" OR "virtual reality therapy" OR "virtual reality exposure therapy" OR "vr exposure" OR "augmented reality" OR "AR" OR "immersive technology" OR "digital intervention" |
| #3 | "Social Skills"[MeSH] OR "social interaction" OR "interpersonal skills" OR "social communication" OR "social competence" OR "Communication" OR "communication skills" OR "verbal skills" OR "nonverbal communication" OR "language development" |
| #4 | #1 AND #2 AND #3 |
| **Embase** | |
| #1 | 'virtual reality'/exp OR 'virtual reality' OR VR OR 'immersive environment' OR 'augmented reality' OR 'mixed reality' OR 'virtual environment*' OR 'computer simulation' OR 'immersive technology' OR 'digital intervention' OR 'virtual reality exposure therapy' OR 'virtual reality therapy' OR 'augmented reality' OR 'AR' |
| #2 | 'autism spectrum disorder'/exp OR 'autism' OR ASD OR 'Asperger syndrome' OR 'pervasive developmental disorder' OR 'childhood disintegrative disorder' OR 'Autistic' OR 'autistic disorder' OR 'asperger syndrome' |
| #3 | 'social skill'/exp OR 'communication skill'/exp OR 'interpersonal communication'/exp OR 'social competence' OR 'social interaction' OR 'verbal communication' OR 'nonverbal communication' OR 'peer interaction' OR 'language development' |
| #4 | #1 AND #2 AND #3 |
| **Cochrane Library** | |
| #1 | MeSH descriptor: [Virtual Reality] explode all trees |
| #2 | (virtual realit*):ti,ab,kw OR (VR):ti,ab,kw OR (virtual environment*):ti,ab,kw OR (immersive environment*):ti,ab,kw OR (virtual simulation):ti,ab,kw |
| #3 | (augmented reality):ti,ab,kw OR (mixed reality):ti,ab,kw OR (computer simulation):ti,ab,kw OR (immersive technolog*):ti,ab,kw OR (virtual reality exposure therapy):ti,ab,kw |
| #4 | (virtual scene*):ti,ab,kw |
| #5 | #1 OR #2 OR #3 OR #4 |
| #6 | MeSH descriptor: [Autism Spectrum Disorder] explode all trees |
| #7 | (autism spectrum disorder*):ti,ab,kw OR (ASD):ti,ab,kw OR (autis*):ti,ab,kw OR (pervasive developmental disorder*):ti,ab,kw OR (autistic disorder*):ti,ab,kw |
| #8 | (pervasive developmental disorder):ti,ab,kw OR (asperger syndrome):ti,ab,kw OR (childhood disintegrative disorder):ti,ab,kw OR (Autistic):ti,ab,kw OR (autism):ti,ab,kw |
| #9 | #6 OR #7 OR #8 |
| #10 | MeSH descriptor: [Social Skills] explode all trees |
| #11 | (social skill*):ti,ab,kw OR (communication skill*):ti,ab,kw OR (social interaction):ti,ab,kw OR (interpersonal skill*):ti,ab,kw OR (conversation skill*):ti,ab,kw |
| #12 | (interpersonal communication):ti,ab,kw OR (social competence):ti,ab,kw OR (social interaction):ti,ab,kw OR (verbal communication):ti,ab,kw OR (nonverbal communication):ti,ab,kw |
| #13 | (language development):ti,ab,kw OR (Communication):ti,ab,kw OR (peer interaction):ti,ab,kw |
| #14 | #10 OR #11 OR #12 OR #13 |
| **Web of Science** | |
| TS=(("virtual realit*" OR "VR" OR "immersive technolog*" OR "immersive environment*" OR "virtual simulation" OR "augmented reality" OR "mixed reality" OR "virtual reality exposure therapy" OR "virtual environment*" OR "vr exposure" OR "digital intervention" OR "AR" ) AND ("autism spectrum disorder*" OR "ASD" OR "pervasive developmental disorder*" OR "autistic disorder*" OR "Autistic" OR "autism" OR "pervasive developmental disorder" OR "asperger syndrome") AND ("social skill*" OR "communication skill*" OR "social interaction" OR "interpersonal skills" OR "social communication" OR "social competence" OR "Communication" OR "communication skills" OR "verbal skills" OR "nonverbal communication" OR "language development")) | |
| **EBSCOhost** | |
| （"Virtual Reality" OR "VR" OR "virtual environment" OR "immersive technology" OR "virtual reality therapy" OR "virtual reality exposure therapy" OR "vr exposure" OR "augmented reality" OR "AR" OR "immersive technology" OR "digital intervention"）AND("Autism Spectrum Disorder" OR "autism" OR "ASD" OR "Autistic" OR "autistic disorder" OR "pervasive developmental disorder" OR "asperger syndrome") AND ("Social Skills" OR "social interaction" OR "interpersonal skills" OR "social communication" OR "social competence" OR "Communication" OR "communication skills" OR "verbal skills" OR "nonverbal communication" OR "language development") | |
| CNKI/WanFang/VIP | |
| #1 | Virtual reality technology + virtual reality +VR+ augmented reality +AR+ spiritual realm technology + virtual |
| #2 | Autism Spectrum disorder + Autism spectrum disorder + autism +ASD |
| #3 | Social skills + communication ability + social interaction + social + communication + society + exchange |
| #4 | #1 AND #2 AND #3 |

**Supplementary 2 Cochrane risk-of-bias assessment results**

**
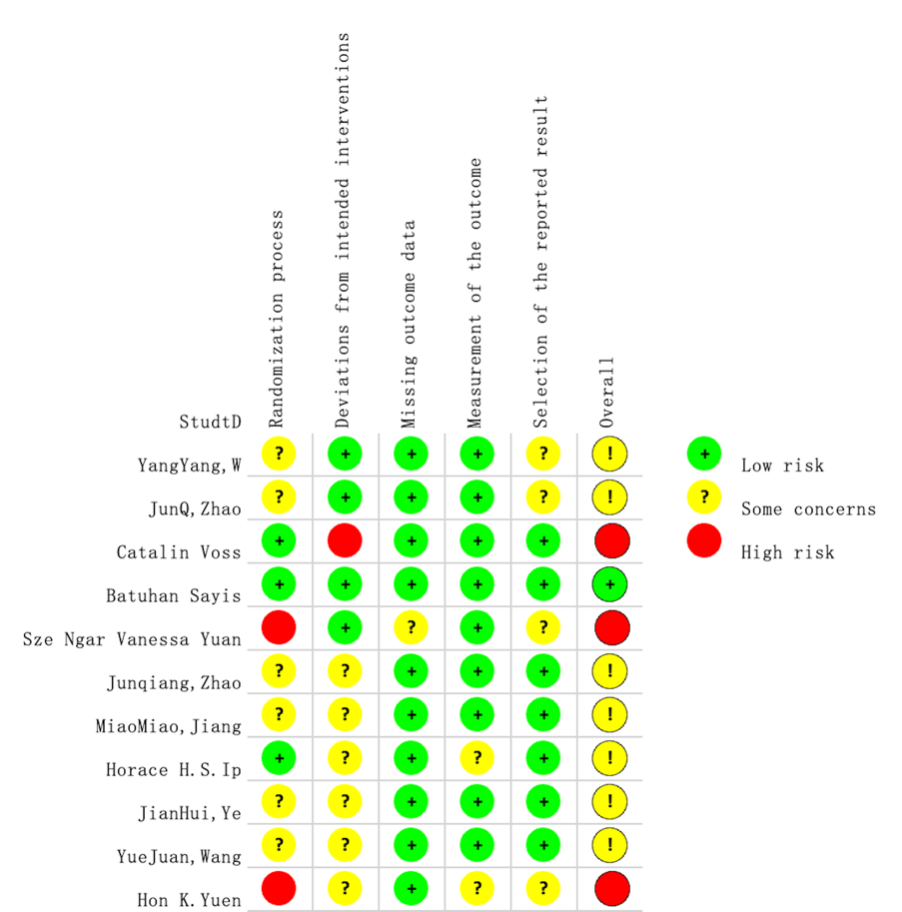
**

**Fig.S3.1 Risk-of-Bias Assessment for Included RCTs Using Revised Cochrane ROB2 Tool**

**
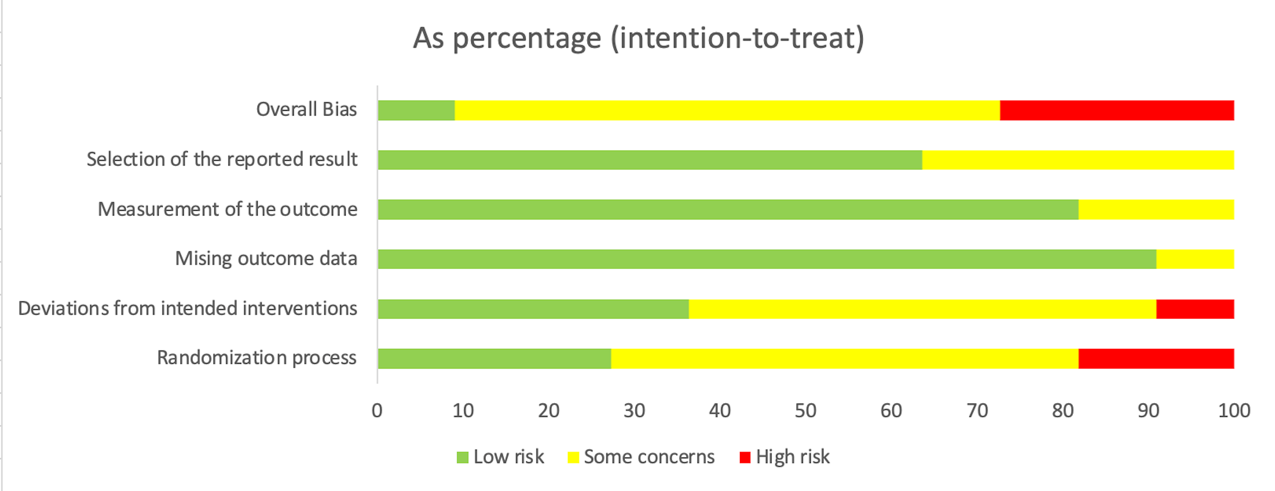
**

**Fig.S3.2 Cochrane risk bias percentile plot**

**Supplementary 3 Exploratory Quantitative Synthesis Findings**

**
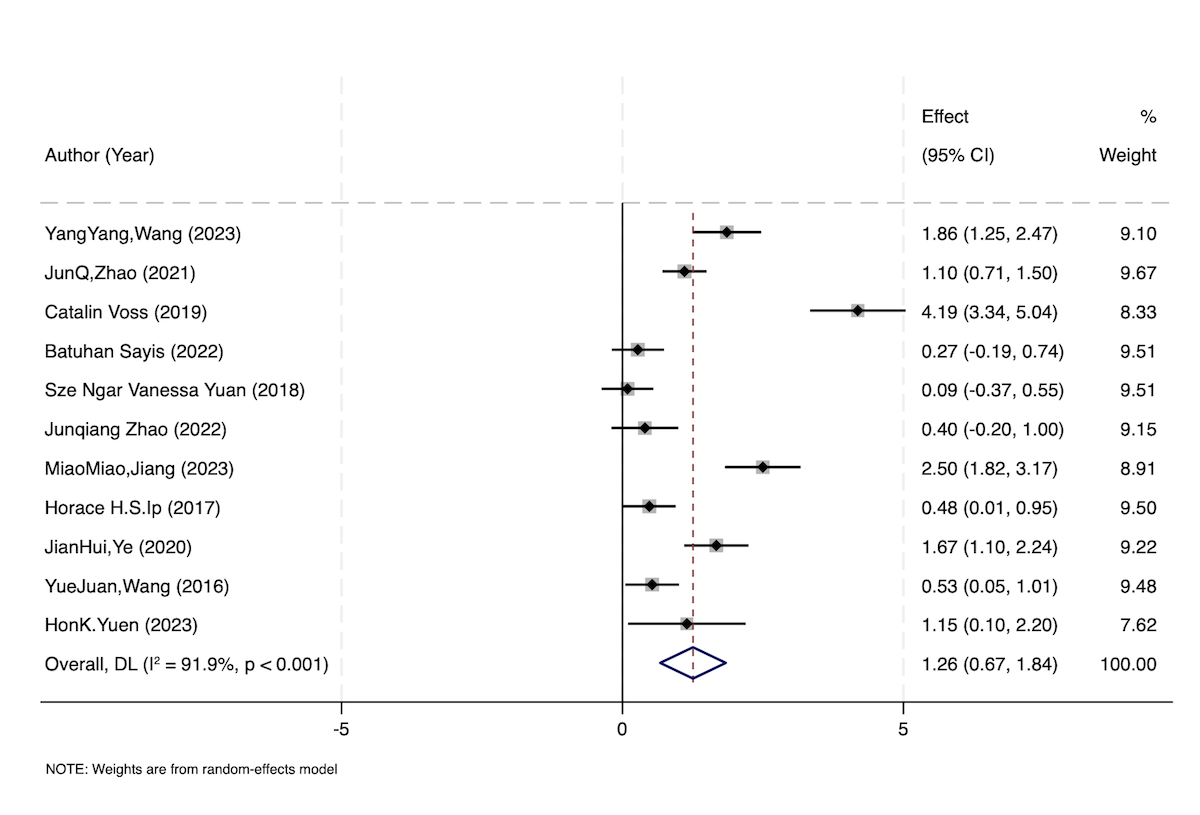
**

**Fig.S4.1 Forest Plot of Exploratory Pairwise Meta-Analysis: Virtual Reality Interventions vs. Control for Social and Communication Skills.**

| **Tab.S4.2 Exploratory Network Meta-Analysis League Table: Standardized Mean Differences (SMD) and 95% Confidence Intervals for All Pairwise Comparisons.** | | | | | | | |
| --- | --- | --- | --- | --- | --- | --- | --- |
| Digitalplatform |  |  |  |  |  |  |  |
| -4.92 (-13.58,3.73) | HMD |  |  |  |  |  |  |
| -2.89 (-14.27,8.49) | 2.03 (-8.77,12.83) | Glass |  |  |  |  |  |
| 3.15 (-8.21,14.51) | 8.07 (-2.71, 18.85) | 6.04 (-7.03,19.11) | MR |  |  |  |  |
| 2.95 (-8.51,14.41) | 7.87 (-3.01,18.76) | 5.84 (-7.32,19.00) | -0.20 (-13.34,12.94) | CAVE |  |  |  |
| 1.45 (-10.04,12.94) | 6.37 (-4.54,17.29) | 4.34 (-8.84,17.52) | -1.70 (-14.87,11.47) | -1.50 (-14.75,11.75) | HCAVE |  |  |
| -4.40 (-14.05,5.25) | 0.53 (-8.44,9.49) | -1.51 (-13.12,10.11) | -7.55 (-19.14,4.05) | -7.35 (-19.04,4.35) | -5.85 (-17.57,5.88) | Computer |  |
| 3.25 (-3.37,9.87) | **8.17 (2.60,13.74)** | 6.14 (-3.11,15.39) | 0.10 (-9.13,9.33) | 0.30 (-9.05,9.65) | 1.80 (-7.59,11.19) | 7.65 (0.63,14.66) | UC |

| **Table S4.3. Surface Under the Cumulative Ranking Curve (SUCRA) Values and Ranks from the Exploratory Network Meta-Analysis.** | | |
| --- | --- | --- |
| Intervention measure | SUCRA | Rank |
| HMD | 82.6 | 1 |
| Computer | 77.9 | 2 |
| Glass | 67.5 | 3 |
| Digital platform | 49.2 | 4 |
| HCAVE | 40.5 | 5 |
| CAVE | 30.4 | 6 |
| MR | 28.9 | 7 |
| UC | 22.9 | 8 |
| ***SUCRA*** The area under the cumulative ranking probability graph of virtual reality intervention. | | |

**
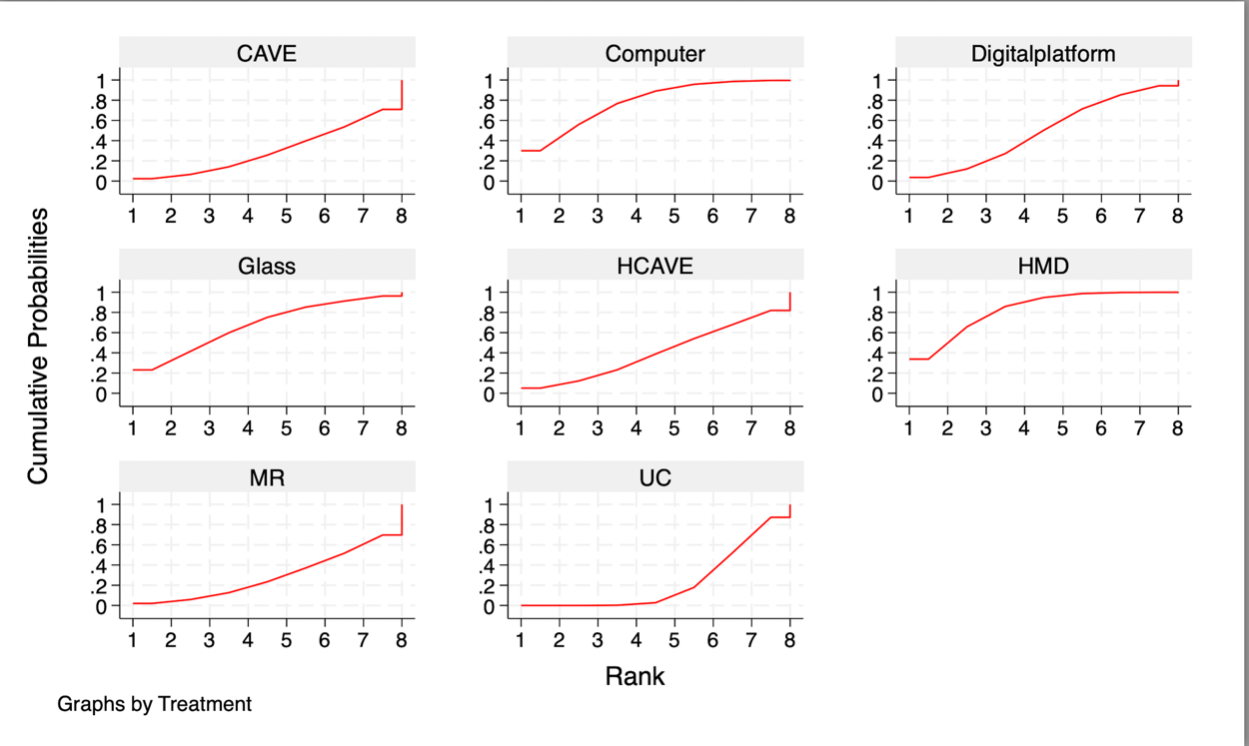
**

**Fig. S4.4 Ranking Plots of VR Interventions Based on SUCRA Probabilities for Social Communication Improvement**

**Supplementary 4 Meta-regression and subgroup analyses**

| **Table S5.1 Meta-regression analysis of Potential Effect Modifiers** | | | | |
| --- | --- | --- | --- | --- |
|  | Coef. (SE) | t | p | 95%CI |
| Region | -0.56(0.96) | -0.06 | 0.96 | (-2.41,2.30) |
| Form | 0.26(0.52) | 0.49 | 0.64 | (-1.01,1.52) |
| Length | 0.21(0.75) | 0.28 | 0.79 | (-1.63,2.04) |
| Duration | -0.40(0.56) | -0.71 | 0.50 | (-1.78,0.98) |

| **Table S5.2 Subgroup analyses of Potential Effect Modifiers** | | | | | | | |
| --- | --- | --- | --- | --- | --- | --- | --- |
|  | n | Hedges’ g | 95% CI | *I^2^* (%) | Test of heterogeneity | | |
|  |  |  |  |  | *Q* | *df* | *p* |
| **Region** |  |  |  |  | 6.56 | 2 | **0.038** |
| Asia | 8 | 1.055 | (0.526,1.585) | 88.1% |  |  |  |
| America | 2 | 2.684 | (-0.297,5.665) | 94.9% |  |  |  |
| Europe | 1 | 0.274 | **(-0.190,0.738)** | . |  |  |  |
| **Form** |  |  |  |  | 65.20 | 3 | **0.000** |
| I-VR | 7 | 0.970 | (0.403,1.536) | 88.4% |  |  |  |
| AR | 1 | 4.189 | **(3.339,5.039)** | . |  |  |  |
| MR | 1 | 0.274 | **(-0.190,0.738)** | . |  |  |  |
| D-VR | 2 | 1.549 | (1.046,2.053) | 0.0% |  |  |  |
| Length(month) |  |  |  |  | 0.29 | 2 | 0.863 |
| ≤1month | 3 | 1.076 | (-0.031,2.183) | 88.0% |  |  |  |
| ≤3month | 4 | 1.556 | (0.035,3.078) | 96.2% |  |  |  |
| ≤6month | 4 | 1.119 | (0.376,1.863) | 89.2% |  |  |  |
| **Duration(min)** |  |  |  |  | 12.26 | 2 | **0.002** |
| ≤20 | 6 | 1.336 | (0.470，2.202) | 93.7% |  |  |  |
| ≤40 | 2 | 2.065 | **(1.253,2.876)** | 70.3% |  |  |  |
| ≤60 | 3 | 0.443 | **(-0.039,0.925)** | 49.1% |  |  |  |

**Supplementary 5 Sensitivity Analyses**

**
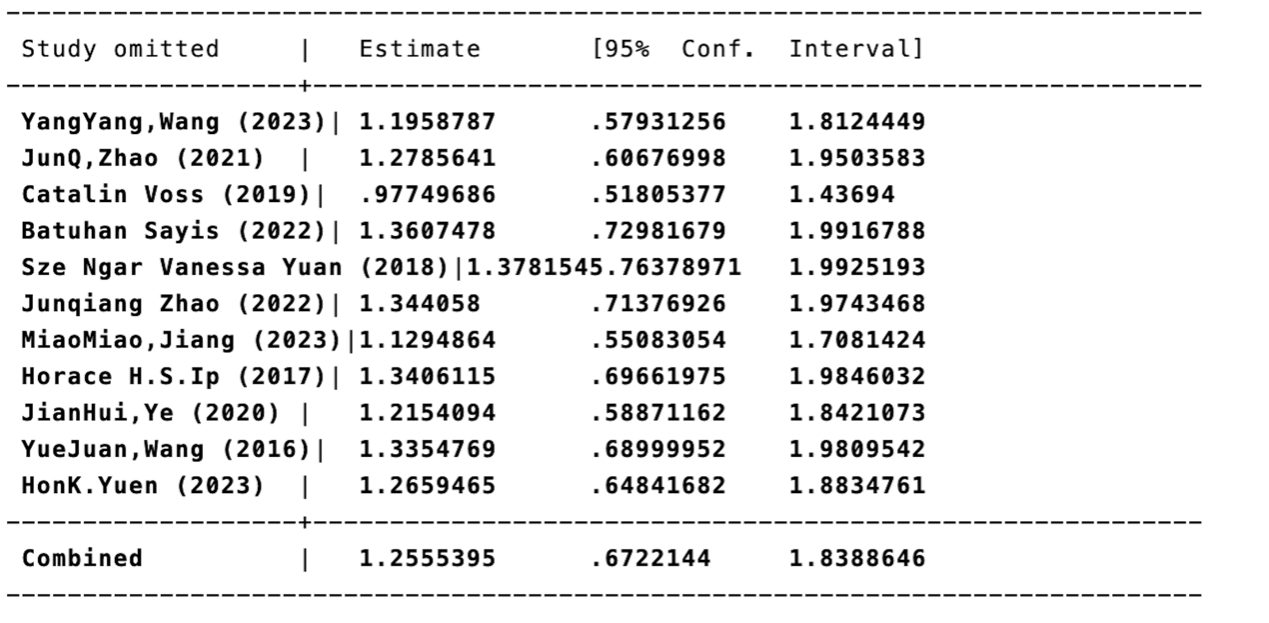
**

**Fig. S6 Sensitivity Analysis by Sequential Exclusion of High Risk-of-Bias Studies**

**Supplementary 6 Comparison-adjusted funnel plot**

**
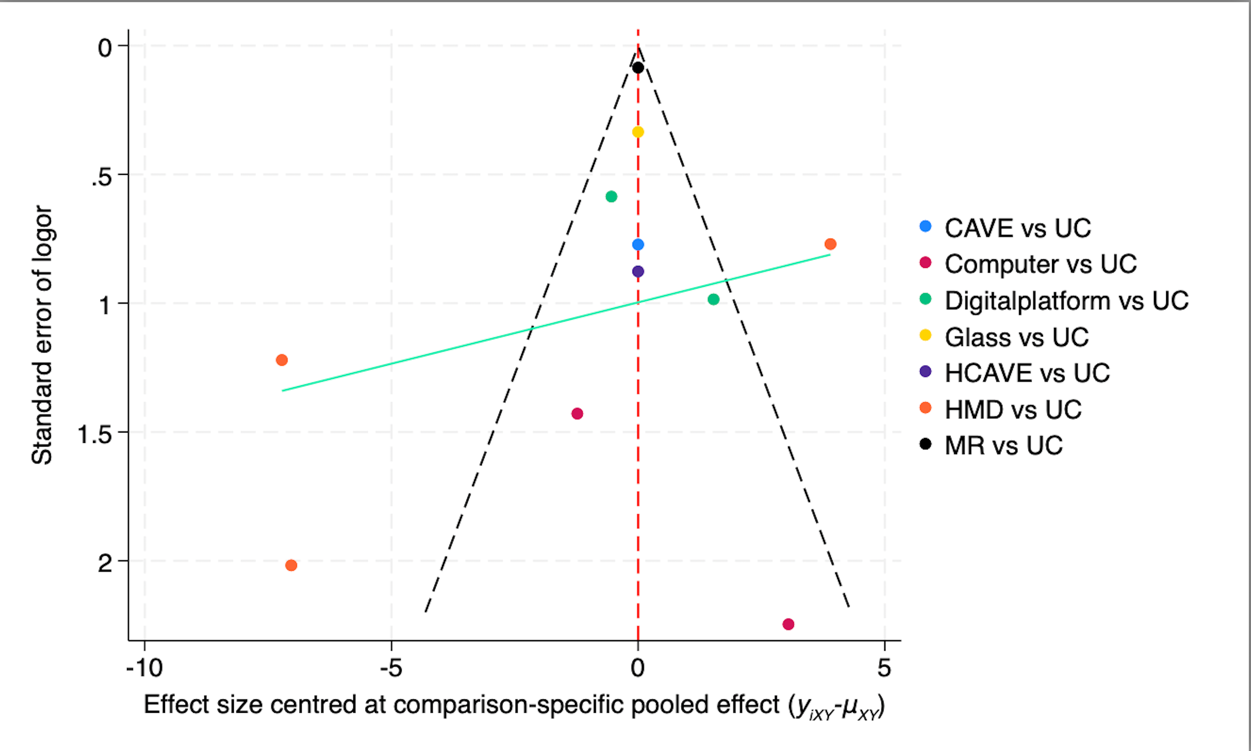
**

**Fig. S7 Comparison-Adjusted Funnel Plot for Assessment of Small-Study Effects**

**Supplementary 7 Grading the evidence of the network meta-analysis using CINeMA**

| **Table S8 Confidence in Network Meta-Analysis (CINeMA) Assessment for Social Communication Outcomes** | | | | | | | | |
| --- | --- | --- | --- | --- | --- | --- | --- | --- |
| **Comparison** | **N of studies** | **Within-study bias** | **Reporting bias** | **Indirectness** | **Imprecision** | **Heterogeneity** | **Incoherence** | **Confidence rating** |
| CAVE:UC | 1 | Major concerns | Low risk | No concerns | Major concerns | No concerns | Some concerns | Very low |
| Computer:UC | 2 | Some concerns | Low risk | No concerns | Some concerns | Some concerns | Some concerns | Low |
| Digital platform:UC | 2 | Some concerns | Low risk | No concerns | Major concerns | No concerns | Some concerns | Low |
| Glasss:UC | 1 | Major concerns | Low risk | No concerns | No concerns | Major concerns | Some concerns | Very low |
| HCAVE:UC | 1 | Some concerns | Low risk | No concerns | Major concerns | No concerns | Some concerns | Low |
| HMD:UC | 3 | Some concerns | Low risk | No concerns | Major concerns | No concerns | Some concerns | Low |
| MR:UC | 1 | No concerns | Low risk | No concerns | Major concerns | No concerns | Some concerns | Low |
| CAVE:Computer | 0 | Major concerns | Low risk | No concerns | Major concerns | No concerns | Some concerns | Very low |
| CAVE:Digital platform | 0 | Some concerns | Low risk | No concerns | Major concerns | No concerns | Some concerns | Low |
| CAVE:Glasss | 0 | Major concerns | Low risk | No concerns | Some concerns | Some concerns | Some concerns | Low |
| CAVE:HCAVE | 0 | Some concerns | Low risk | No concerns | Major concerns | No concerns | Some concerns | Low |
| CAVE:HMD | 0 | Some concerns | Low risk | No concerns | Major concerns | No concerns | Some concerns | Low |
| CAVE:MR | 0 | No concerns | Low risk | No concerns | Major concerns | No concerns | Some concerns | Low |
| Computer:Digital platform | 0 | Some concerns | Low risk | No concerns | Major concerns | No concerns | Some concerns | Low |
| Computer:Glasss | 0 | Major concerns | Low risk | No concerns | Major concerns | No concerns | Some concerns | Very low |
| Computer:HCAVE | 0 | Some concerns | Low risk | No concerns | Major concerns | No concerns | Some concerns | Low |
| Computer:HMD | 0 | Some concerns | Low risk | No concerns | Major concerns | No concerns | Some concerns | Low |
| Computer:MR | 0 | No concerns | Low risk | No concerns | Major concerns | No concerns | Some concerns | Low |
| Digital platform:Glasss | 0 | Some concerns | Low risk | No concerns | Some concerns | Some concerns | Some concerns | Low |
| Digital platform:HCAVE | 0 | Some concerns | Low risk | No concerns | Major concerns | No concerns | Some concerns | Low |
| Digital platform:HMD | 0 | Some concerns | Low risk | No concerns | Major concerns | No concerns | Some concerns | Low |
| Digital platform:MR | 0 | No concerns | Low risk | No concerns | Major concerns | No concerns | Some concerns | Low |
| Glasss:HCAVE | 0 | Some concerns | Low risk | No concerns | Some concerns | Some concerns | Some concerns | Low |
| Glasss:HMD | 0 | Some concerns | Low risk | No concerns | No concerns | Major concerns | Some concerns | Low |
| Glasss:MR | 0 | No concerns | Low risk | No concerns | Some concerns | Some concerns | Some concerns | Low |
| HCAVE:HMD | 0 | Some concerns | Low risk | No concerns | Major concerns | No concerns | Some concerns | Low |
| HCAVE:MR | 0 | No concerns | Low risk | No concerns | Major concerns | No concerns | Some concerns | Low |
| HMD:MR | 0 | No concerns | Low risk | No concerns | Major concerns | No concerns | Some concerns | Low |
